# Cannabis Use Is Associated With an Increased Risk of Intestinal Obstruction in Patients Hospitalized With Diverticulitis

**DOI:** 10.7759/cureus.16768

**Published:** 2021-07-30

**Authors:** Daniel Rim, Alexander Kaye, Akash Ranpura, Siddharth Verma

**Affiliations:** 1 Internal Medicine, Rutgers University, Newark, USA; 2 Gastroenterology and Hepatology, Veterans Affairs Medical Center, East Orange, USA

**Keywords:** diverticulitis, diverticular disease, cannabis, intestinal obstruction, patient outcomes

## Abstract

Objectives

Diverticulitis is a common cause of hospitalization. The use of substances such as tobacco and alcohol can predispose patients to diverticulitis, and smoking is also associated with an increased risk of diverticulitis complications. Cannabis availability is growing in the United States, but there is a lack of data on the effects of cannabis use on the outcomes of diverticulitis. Thus, we investigated the effects of cannabis use on diverticulitis outcomes.

Methods

A retrospective analysis was conducted using 2014 data from the National Inpatient Sample. Patient demographics and outcomes of diverticulitis were compared between the groups with and without a history of cannabis use. The outcomes of interest were inpatient mortality, length of stay, total hospital charge, intestinal obstruction, shock/hypotension, colectomy, intestinal abscess, intestinal fistula, and intestinal perforation.

Results

Among 48,214 patients with diverticulitis, 447 patients had a history of cannabis use. Patients with a history of cannabis use were younger, more likely to be male, less likely to be White, had a lower Charlson Comorbidity Index, and had shorter hospital stays. There were no significant differences in inpatient mortality and total hospital charge. After adjusting for age, sex, race, and the Charlson Comorbidity Index, cannabis use was an independent risk factor for intestinal obstruction in patients hospitalized with diverticulitis. There were no statistically significant differences in other outcomes.

Conclusions

This study indicates that patients hospitalized with diverticulitis with a history of cannabis use are more likely to have an intestinal obstruction. Inhibition of gastrointestinal motility by cannabis in the setting of diverticular inflammation may explain this finding.

## Introduction

Diverticulitis is a well-known colonic pathology that is caused by the inflammation or infection of the diverticula. Diverticular disease is common with a prevalence as high as 65% in patients aged 85 years; about 10-25% of these patients develop the symptomatic disease at some point [1,2]. Several non-modifiable risk factors associated with diverticulitis include older age, male sex, and certain genetic syndromes such as Marfan and Ehlers-Danlos [3].

Notably, the lifestyle of patients plays an important role in the likelihood of developing an episode of diverticulitis. Some modifiable risk factors that may increase the risk of diverticulosis and diverticulitis include red meat consumption, certain medications such as steroids and opiates, and specific comorbidities such as obesity, hypertension, hypothyroidism, and type 2 diabetes [3,4]. Non-medicinal substances can also impact a patient’s likelihood of developing diverticulitis. Smoking is a well-known independent risk factor for the development of diverticulitis [5]. In addition to an increased incidence of diverticulitis, smoking has been associated with an increased rate of complications and longer hospital stays for diverticulitis patients [6]. In the younger adult population, chronic alcohol use can also increase the likelihood of an episode of diverticulitis, and alcohol use, in general, is associated with an increased rate of diverticulitis recurrence [3,7,8]. Despite the popularity of cannabis, there is currently little data exploring the connection between the use of this substance and how it impacts the risk of developing or the severity of diverticulitis.

Cannabis has recently become more readily available to the general population. As of May 2021, 36 states have approved the use of cannabis for specific medical indications, and 17 states and Washington D.C. have legalized the use of recreational marijuana as well [9]. Cannabis can impact gastric emptying and colonic motility due to the cannabinoid receptors found along the gastrointestinal tract [10]. More specifically, activation of the cannabinoid receptors is known to cause inhibition of peristalsis, gastric acid secretion, and decreased colonic motility [11]. Furthermore, cannabis can cause gastrointestinal dysmotility [10]. Given that cannabis is growing in availability and its known impact on gastrointestinal tract motility, it is important to understand better how this substance may impact diverticulitis. Therefore, this study further explores the outcomes of patients with diverticulitis who have a history of cannabis use at a national level.

This article was previously presented as a poster at the Annual Department of Medicine Research Day on May 27, 2021.

## Materials and methods

This is a retrospective cohort study using data from the National Inpatient Sample (NIS), 2014, Healthcare Cost and Utilization Project (HCUP), and Agency for Healthcare Research and Quality, which is known as the largest all-payer inpatient database in the United States [12]. Adult patients aged 18 years and above hospitalized with diverticulitis were included in the study. The International Classification of Diseases-Ninth Edition Revision, Clinical Modification (ICD-9 CM) codes were used to identify diagnoses in this database. Patients were then divided into two groups: those with and without a history of cannabis use. Patient demographics and characteristics including age, sex, race, and the Charlson Comorbidity Index were compared between the groups. The Charlson Comorbidity Index is a tool frequently used for comorbidity adjustment for confounding [13,14]. The clinical outcomes of diverticulitis including inpatient mortality, length of stay, total hospital charge, hypotension/shock, intestinal abscess, intestinal obstruction, intestinal fistula, intestinal perforation, and colectomy were compared.

SPSS version 26.0 (Armonk, NY: IBM Corp) was used for statistical analyses. Chi-squared tests and independent t-tests were used to compare proportions and means, respectively. Statistical analyses performed in this study were two-tailed, and a p-value less than 0.05 was considered statistically significant. Multivariate logistic regression analysis was performed to determine if cannabis use is an independent predictor for the clinical outcomes, adjusting for age, sex, race, and the Charlson Comorbidity Index.

## Results

Among 48,214 patients with diverticulitis identified in the study, 447 patients had a history of cannabis use. Patient demographics, characteristics, length of stay, inpatient mortality, and total hospital charge are demonstrated in Table 1. Among patients hospitalized with diverticulitis, those with cannabis use were younger (45.8 vs 60.6, p < 0.05), more likely to be male (74.5% vs. 41.5%, p < 0.05), less likely to be White (60.1% vs. 77.3%, p < 0.05), had lower Charlson Comorbidity Index (1.1 vs. 2.5, p < 0.05), and had shorter length of stay (4.3 days vs. 4.7 days, p < 0.05). There were no statistically significant differences in inpatient mortality (0% vs. 0.4%, p = 0.20) and total hospital charge ($36,573 vs. $38,568, p = 0.45).

**Table 1:** Demographics, characteristics, length of stay, total hospital charge, and inpatient mortality among diverticulitis patients with and without a history of cannabis use *Exact numbers are not included in the table due to small sample sizes.

Variable	With cannabis use	Without cannabis use	p-Value
N = 48,214	N = 447	N = 47,767	
Patient age, mean (SD)	45.8 (11.4)	60.6 (15.3)	<0.05
Sex, N (%)			<0.05
Female	114 (25.5%)	27,913 (58.5%)	
Male	333 (74.5%)	19,827 (41.5%)	
Race, N (%)			<0.05
White	262 (60.1%)	35,323 (77.3%)	
Black	107 (24.5%)	3711 (8.1%)	
Hispanic	51 (11.7%)	4986 (10.9%)	
Asian or Pacific Islander	*	426 (0.9%)	
Native American	*	177 (0.4%)	
Other	*	1052 (2.3%)	
Length of stay, in days (SD)	4.3 (3.9)	4.7 (4.4)	<0.05
Total hospital charges, in $ (SD)	36,573 (56,238)	38,568 (54,259)	0.45
Inpatient mortality	0 (0%)	178 (0.4%)	0.20
Charlson Comorbidity Index, mean (SD)	1.1 (1.4)	2.5 (2.1)	<0.05

Table 2 compares clinical outcomes of diverticulitis in groups with and without a history of cannabis use. Patients with a history of cannabis use had a higher prevalence of intestinal abscess (19.5% vs. 15.2%, p < 0.05). There were no statistically significant differences in the prevalence of shock/hypotension, colectomy, intestinal obstruction, intestinal fistula, or intestinal perforation. Due to small sample sizes of shock/hypotension, intestinal fistula, and intestinal perforation, further analysis of these outcomes was not performed.

**Table 2:** Clinical outcomes among diverticulitis patients with and without a history of cannabis use *Exact numbers are not included in the table due to small sample sizes; values are reported as numbers (%).

Outcomes	With cannabis use	Without cannabis use	p-Value
Shock/hypotension	*	204 (0.4%)	0.95
Colectomy	47 (10.5%)	5865 (12.3%)	0.26
Intestinal abscess	87 (19.5%)	7281 (15.2%)	<0.05
Intestinal obstruction	19 (4.3%)	1572 (3.3%)	0.26
Intestinal fistula	*	437 (0.9%)	0.30
Intestinal perforation	*	153 (0.3%)	0.72

Table 3 shows odds ratios of clinical outcomes after adjusting for age, sex, race, and the Charlson Comorbidity Index. Cannabis use was an independent risk factor for intestinal obstruction (adjusted odds ratio {aOR} 1.69, 95% confidence interval {CI}: 1.05-2.73, p < 0.05). However, adjusted odds ratios of colectomy (aOR 0.83, 95% CI: 0.61-1.13, p = 0.23) and intestinal abscess (aOR 1.20, 95% CI: 0.95-1.53, p = 0.13) were not statistically significant.

**Table 3:** Multivariate logistic regression analysis of clinical outcomes *Adjusted for age, sex, race, and the Charlson Comorbidity Index.

Outcomes	Adjusted odds ratio* (95% CI)	p-Value
Colectomy	0.83 (0.61-1.13)	0.23
Intestinal abscess	1.20 (0.95-1.53)	0.13
Intestinal obstruction	1.69 (1.05-2.73)	<0.05

## Discussion

Prior to this study, it was established that cannabis could cause gastric dysmotility through its activation of cannabinoid receptors found along the gastrointestinal tract. Despite cannabis’ known impact on gastric emptying and motility, it was unclear how cannabis affects the disease course of diverticulitis. This study showed how cannabis use can affect the outcomes of diverticulitis. As shown above, these data demonstrate that patients hospitalized with diverticulitis with a history of cannabis use have an increased rate of intestinal obstruction. This is an important finding as intestinal obstruction carries with it an elevated mortality risk [15]. While increased mortality was not appreciated in this study, given that marijuana use is becoming increasingly more widespread and popular, this is a consideration that primary care providers should counsel their patients on. In addition, at the population level, anticipating an increased frequency of intestinal obstruction cases secondary to cannabis use, there will be an increased economic burden on the healthcare system. The increased number of visits to primary care physicians and gastroenterologists for evaluation of symptoms caused by an intestinal obstruction in cannabis users is also anticipated.

Despite the increased risk of intestinal obstruction, the use of cannabis in diverticulitis patients did not affect rates of other outcomes. These findings can be explained by the role of cannabinoid receptors located in the colon. Through activation of the cannabinoid receptors, colonic motility is decreased, which may be the primary instigator of the intestinal obstruction [11,16]. While colonic motility is decreased, some data indicate a potential protective effect that cannabinoids may provide. There is evidence in animal models supporting a protective role of cannabinoids in the setting of inflammatory bowel disease by decreasing inflammatory damage and diarrhea [11,17]. As diverticulitis is also a state of intestinal inflammation, it is possible to extrapolate that cannabis use should not worsen the outcomes related to inflammation, such as colectomy and intestinal abscess.

This study has several limitations. Regarding the diagnosis of cannabis use, ICD-9 coding only allows for the indication of whether the patient is using a cannabis product without a quantitative measurement. Without the ability to quantify how much cannabis a patient with diverticulitis may be using, it is difficult to assess if the risk of intestinal obstruction is dose-dependent or if the risk of intestinal obstruction is the same regardless of the amount of cannabis used.

This analysis is also limited by the functionality of performing research with the NIS database. NIS relies on accurate billing code inputs by healthcare providers. Inaccurate billing can result in the frequency of diverticulitis, cannabis use, and the risk for complications being over or underrepresented. The illegality of cannabis use in many states in the United States in previous years may have contributed to underreporting of its use. An additional limitation of NIS is that it only represents patients that have been hospitalized. Under 20% of patients who present for evaluation of diverticular disease require hospitalization, indicating that the results of this study may not represent those who are not hospitalized [18]. Despite these limitations, one of the greatest strengths of this study is the large sample size, which allows the estimation of patient characteristics and outcomes at a national level. In addition, this study was bolstered by the multivariate logistic regression analysis that adjusted for potential confounding factors.

## Conclusions

In summary, patients with a history of cannabis use who develop diverticulitis are at increased risk for intestinal obstruction, while its use does not seem to play a significant role in altering other outcomes or the prognosis of patients suffering from diverticulitis. This study will become more relevant as more and more states legalize recreational cannabis, thus increasing the access and its use and provides data to better counsel patients about cannabis use. Additional research into the therapeutic aspects as well as adverse events from the use of cannabis is necessary to meet the increased use of this substance among the adult patient population.
